# Supplementary material for: A Highly Effective African Swine Fever Virus Vaccine Elicits a Memory T Cell Response in Vaccinated Swine
Source: Pathogens. 2022 Nov 29;11(12):1438. doi: 10.3390/pathogens11121438 (PMC9783822; doi:10.3390/pathogens11121438)
Supplement: Supplementary file 1 [file pathogens-11-01438-s001.zip › pathogens-1830988-supplementary/Supplemental material/Supplemental Tables Final.pdf]

Supplemental Table S1. Memory T Cell Panel antibody and fluorophore list.

Channel (Bandpass Filter)	*Antibody (clone)*	*Antibody Manufacturer (CAT#)*	*Fluorophore Conjugate (if not pre-conjugated)*	*Fluorophore Conjugation kits Manufacturer (Cat#)*
FITC (530/30)	Mouse anti-pig CD3ε-FITC (BB23-8E6-8C8)	BD (559582)	NA	NA
PE (572/28)	Mouse anti-bovine IFNγ-RPE (CC302)	Bio-Rad (MCA1783PE)	NA	NA
PE-Texas Red (615/20)	Mouse anti-bovine CD62L (CC32)	Bio-Rad (MCA1649G)	PE-Texas Red	Abcam (ab269899)
PerCP (675/30)	Rat anti-human CD197-PerCP-eFLuor710 (3D12)	Invitrogen (46-1979-42)	NA	NA
PEcy7 (780/60)	Mouse anti-pig CD4α-PEcy7 (74-12-4)	BD (561473)	NA	NA
APC (675/30)	Mouse anti-pig CD8α-AlexaFluor647 (76-2-11)	BD (561475)	NA	NA
Pacific Blue (445/45)	Rat anti-human CD44-eFluor450 (IM7)	Invitrogen (48-0441-80)	NA	NA
Pacific Orange (572/28)	LIVE/DEAD Fixable Yellow	Invitrogen (L34959)	NA	NA
AmCyan (530/30)	Mouse anti-pig CD25 (K231.3B2)	Bio-Rad (MCA1736GA)	QDot565	Invitrogen (S10450)
Qdot 800 (780/60)	Mouse anti-pig CD27 (B30C7)	Bio-Rad (MCA5973GA)	QDot800	Invitrogen (S10455)

Supplemental Table S2. Regulatory T and γδT Cell Panel antibody and fluorophore list.

Channel (Bandpass Filter)	*Antibody (clone)*	*Antibody Manufacturer (Cat#)*	*Fluorophore Conjugate (if not pre-conjugated)*	*Fluorophore Conjugation kit Manufacturer (Cat#)*
FITC (530/30)	Mouse anti-pig CD8β-FITC (PPT23)	Bio-Rad (MCA5954F)	NA	NA
PE (572/28)	Mouse anti-bovine IFNγ-RPE (CC302)	Bio-Rad (MCA1783PE)	NA	NA
PE-Texas Red (615/20)	Mouse anti-pig TCR1δ-chain (PGBL22A)	KingFisher (WS0621S-100)	PE-Texas Red	Abcam (ab269899)
PerCP (675/30)	Mouse anti-pig CD4α (MIL17)	Bio-Rad (MCA1749GA)	PerCPcy5.5	Abcam (ab102911)
PEcy7 (780/60)	Mouse anti-pig CD3 (PPT3)	Bio-Rad (MCA5951GA)	PEcy7	Abcam (ab274150)
APC (675/30)	Rat anti-mouse FoxP3-APC (FJK-16s)	Invitrogen (17-5773-82)	NA	NA
APCcy7 (780/60)	Mouse anti-pig CD25 (K231.3B2)	Bio-Rad (MCA1736GA)	APCcy7	Abcam (ab274159)
Pacific Orange (572/28)	LIVE/DEAD Fixable Yellow	Invitrogen (L34959)	NA	NA

Supplemental Table S3. Myeloid Panel antibodies and fluorophores.

Channel (Bandpass Filter)	*Antibody (clone)*	*Antibody Manufacturer (Cat#)*	*Fluorophore Conjugate (if not pre-conjugated)*	*Fluorophore Conjugation kit Manufacturer (Cat#)*
FITC (530/30)	Mouse anti-pig CD172a-FITC (BL1H7)	Bio-Rad (MCA2312F)	NA	NA
PE (572/28)	Mouse anti-human CD14-PE (REA641)	Miltenyi Biotec (130-113-147)	NA	NA
PE-Texas Red (615/20)	Mouse anti-pig SLAII (2E9/13)	Bio-Rad (MCA2314GA)	PE-Texas Red	Abcam (ab269899)
PerCP (675/30)	Mouse anti-pig CD4α (MIL17)	Bio-Rad (MCA1749GA)	PerCPcy5.5	Abcam (ab102911)
PEcy7 (780/60)	Mouse anti-pig CD3 (PPT3)	Bio-Rad (MCA5951GA)	PEcy7	Abcam (ab274150)
Pacific Orange (572/28)	LIVE/DEAD Fixable Yellow	Invitrogen (L34959)	NA	NA

Supplemental Table S4. Myeloid cell populations analysis after inoculation. Mean ± standard deviation of the change in population percentage of parent or mean fluorescence intensity between *ex vivo* unstimulated and stimulated wells for post-inoculation time points. n=2-10/treatment group/time point. [a]p-value<0.05 compared to within-group baseline (0 dpi). [b]p-value<0.05 compared to time-matched control treatment group.

[*] CD3-CD14-CD172a+SLA-II+.

[**] CD3-CD14-CD172a+SLA-II+CD4-.

[***] CD3-CD14-CD172a+SLA-II+.CD4+.

[****] CD3-CD14+.

	Mock					*ΔI177L*				
	0 dpi	4 dpi	7 dpi	14 dpi	28 dpi	0 dpi	4 dpi	7 dpi	14 dpi	28 dpi
Dendritic Cells (DC-like)[*]	1.11 ± 0.81	0.41 ± 0.26	0.21 ± 0.20	**0.15 ± 0.03**[a]	0.70 ± 0.27	0.58 ± 0.57	0.30 ± 0.39	0.09 ± 0.17	0.55 ± 0.87	0.47 ± 0.23
Conventional DC-like[**]	5.77 ± 2.44	**-0.62 ± 2.74**[a]	0.83 ± 0.47	1.14 ± 1.48	2.69 ± 3.56	4.58 ± 4.28	2.45 ± 1.77	2.75 ± 5.92	-2.12 ± 3.28a	1.52 ± 1.90
SLA-II MFI	8996 ± 8575	23433 ± 3205	25220 ± 2632	10929 ± 2632	-1852 ± 13195	-37406 ± 7469	6184 ± 8537	4856 ± 13254	**17923 ± 13636**[a]	-7153 ± 17044
CD172a MFI	-7431 ± 10037	-514 ± 491	-5 ± 351	-8 ± 455	-13996 ± 4166	-7889 ± 10534	-1704 ± 1339	179 ± 1389	-479 ± 1081	-11167 ± 8594
Plasmacytoid DC-like[***]	-5.24 ± 1.89	-0.21 ± 2.36	-2.35 ± 1.80	-1.31 ± 2.11	-2.34 ± 3.63	-3.59 ± 4.48	-2.28 ± 1.44	-2.38 ± 4.80	**1.81 ± 2.68**[a]	-1.39 ± 2.15
SLA-II MFI	-15420 ± 21331	16117 ± 28477	-17467 ± 111037	-94551 ± 58914	-18460 ± 29227	18310 ± 25121	-50075 ± 90067	32573 ± 44505	-10606 ± 82471	-2302 ± 39664
CD172a MFI	-6026 ± 8023	-591 ± 402	945 ± 1342	-1517 ± 686	-5755 ± 7320	-2569 ± 7323	-2820 ± 2991	286 ± 762	262 ± 1082	-3443 ± 12094
Monocytes[****]	0.48 ± 0.38	-0.08 ± 0.30	0.06 ± 0.09	0.27 ± 0.13	0.004 ± 0.33	0.45 ± 0.47	**-0.65 ± 0.75**[a]	0.21 ± 0.80	**-1.00 ± 1.06**[a,b]	**-0.71 ± 0.47**[a]
SLA-II MFI	-33739 ± 13338	**1075 ± 4240**[a]	**337 ± 3376**[a]	**-5802 ± 7131**[a]	-12333 ± 6709	-26953 ± 28550	**-1660 ± 4432**[a]	**7586 ± 8358**[a]	**3961 ± 3045**[a]	**2717 ± 9141**[a]
CD14	-2202 ± 11609	10681 ± 15640	7476 ± 1150	1750 ± 3411	-2287 ± 3498	-432 ± 13908	11224 ± 9849	-4333 ± 7145	**-19291 ± 7689**[a,b]	**-17156 ± 7678**[a]
CD172a	-2943 ± 5136	-819 ± 1138	**-232263 ± 323163**[a]	-3239 ± 1833	-596 ± 2058	-526 ± 7546	923 ± 3385	**6518 ± 41067**[b]	-1731 ± 5050	-32619 ± 6857

Supplemental Table S5. Myeloid cell populations analysis after challenge. Mean ± standard deviation of the change in population percentage or mean fluorescence intensity between *ex vivo* unstimulated and stimulated wells for post-challenge time points. n=2-6/treatment group/time point. $_{a}$p-value<0.05 compared to within-group baseline (0 dpc). $_{b}$p-value<0.05 compared to time-matched control treatment group.

* CD3-CD14-CD172a+SLA-II+.

** CD3-CD14-CD172a+SLA-II+CD4-.

*** CD3-CD14-CD172a+SLA-II+.CD4+.

**** CD3-CD14+.

	Mock				*ΔI177L*			
	0 dpc	4 dpc	7 dpc	14 dpc	0 dpc	4 dpc	7 dpc	14 dpc
*Dendritic Cells (DC-like)**	0.70 ± 0.27	0.23 ± 0.29	0.87 ± 1.73	*NA*	0.47 ± 0.23	0.07 ± 0.04	0.17 ± 0.11	0.018 ± 0.08
*Conventional DC-like***	2.69 ± 3.56	2.85 ± 1.82	0.39 ± 1.24	*NA*	1.52 ± 1.90	1.74 ± 5.30	2.25 ± 4.40	-1.43 ± 1.23
SLA-II MFI	-1852 ± 13195	-3792 ± 10512	-303 ± 2667	*NA*	-7153 ± 17044	-5069 ± 11862	-6411 ± 25076	-159 ± 9164
CD172a MFI	-13996 ± 4166	-2224 ± 1625	1448 ± 3182	*NA*	-11167 ± 8594	457 ± 1087	-15950 ± 23895	-1189 ± 2517
*Plasmacytoid DC-like****	-2.34 ± 3.65	-1.85 ± 0.12	-0.61 ± 1.44	*NA*	-1.39 ± 2.15	-1.98 ± 2.64	-2.83 ± 4.00	1.22 ± 1.57
SLA-II MFI	-18460 ± 29227	34493 ± 47903	31203 ± 5750	*NA*	-2302 ± 39664	-3064 ± 41212	-7126 ± 38408	-41243 ± 80082
CD172a MFI	-5755 ± 7320	2115 ± 5186	-8264 ± 5633	*NA*	-3444 ± 12094	4088 ± 7177	-11075 ± 14638	-2867 ± 6051
*Monocytes*****	0.004 ± 0.33	-0.02 ± 0.03	0.20 ± 0.38	*NA*	**-0.71 ± 0.47**^{b}	-0.04 ± 0.02	-0.20 ± 0.68	0.005 ± 0.05
SLA-II MFI	-12333 ± 6709	**10099 ± 17461**^{a}	6516 ± 521	*NA*	2717 ± 9141	22300 ± 12584	872 ± 10206	41221 ± 34173
CD14	-2287 ± 3498	1510 ± 2959	-2976 ± 9825	*NA*	**-17156 ± 7678**^{b}	-12513 ± 2809	**-20007 ± 5670**^{b}	**1760 ± 21888**^{a}
CD172a	-596 ± 2058	50830 ± 176447	-37894 ± 14652	*NA*	-32619 ± 6857	65071 ± 171653	-30155 ± 4585	-85021 ± 104930
