# Supplementary figures and images for: A Highly Effective African Swine Fever Virus Vaccine Elicits a Memory T Cell Response in Vaccinated Swine
Source: Pathogens. 2022 Nov 29;11(12):1438. doi: 10.3390/pathogens11121438 (PMC9783822; doi:10.3390/pathogens11121438)

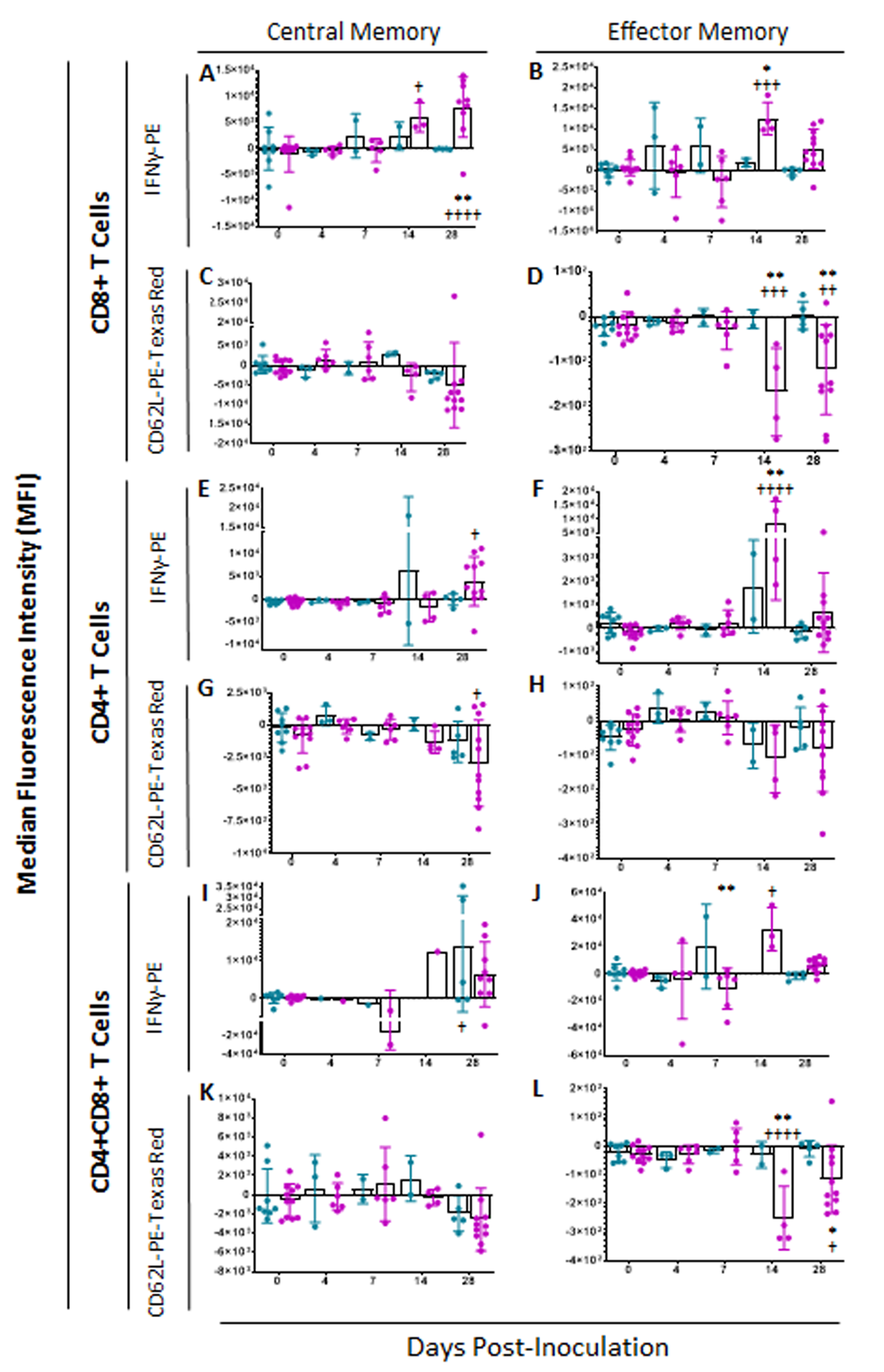

Supplement: Supplementary file 1 [file pathogens-11-01438-s001.zip › pathogens-1830988-supplementary/Supplemental material/Supplemental Figure S1 11.28.22.tif]

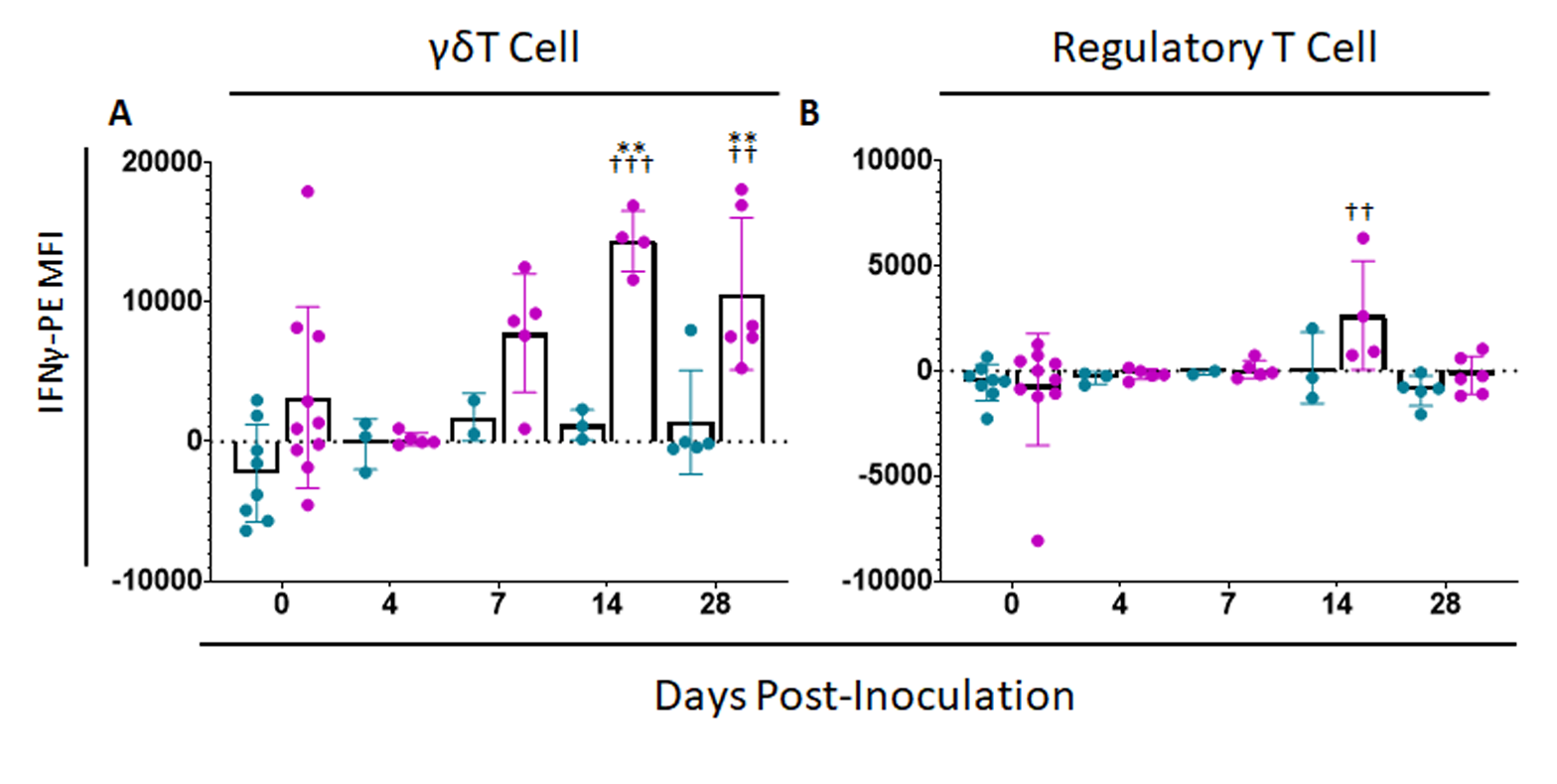

Supplement: Supplementary file 1 [file pathogens-11-01438-s001.zip › pathogens-1830988-supplementary/Supplemental material/Supplemental Figure S2 11.28.22.tif]

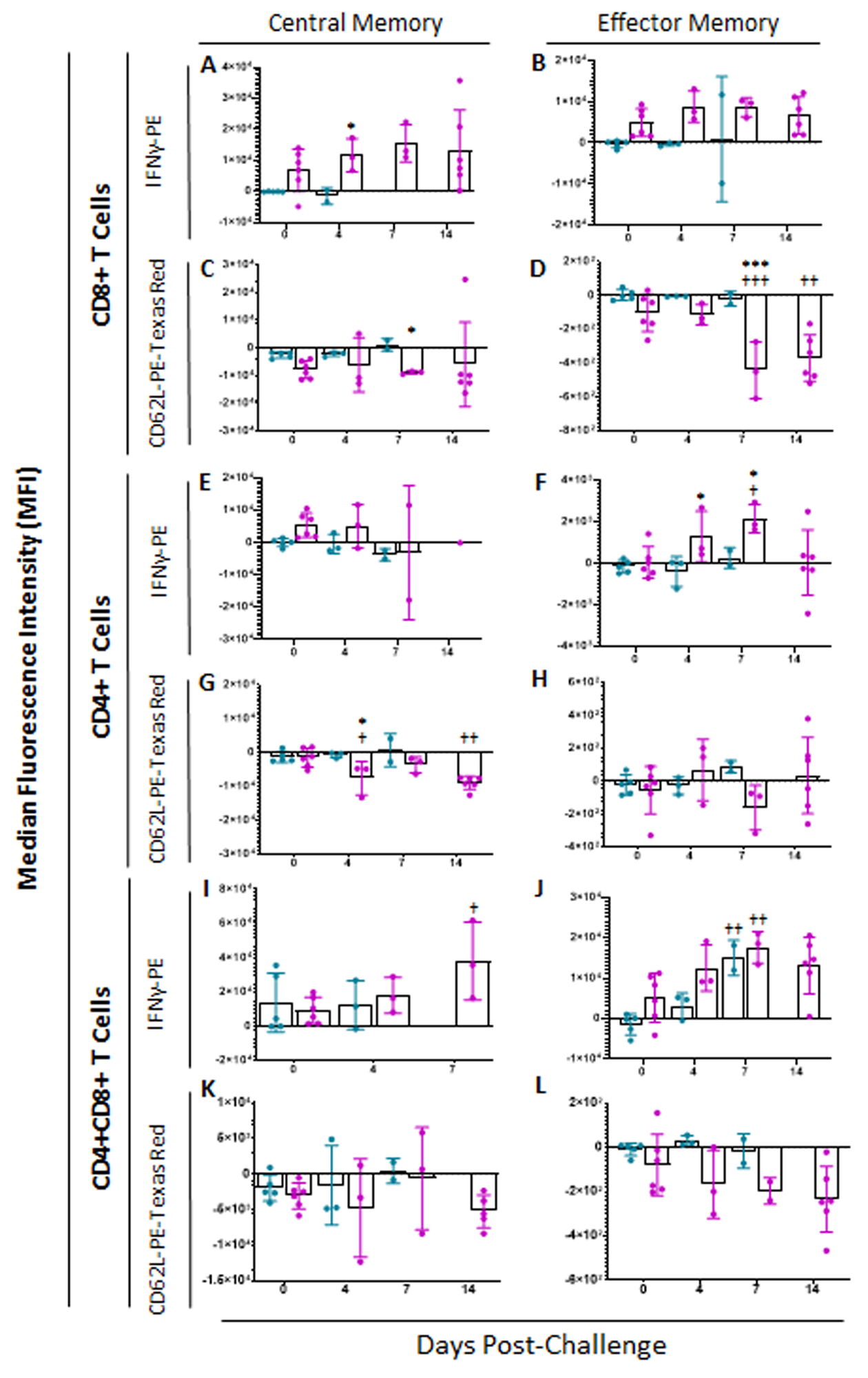

Supplement: Supplementary file 1 [file pathogens-11-01438-s001.zip › pathogens-1830988-supplementary/Supplemental material/Supplemental Figure S3 11.28.22.tif]

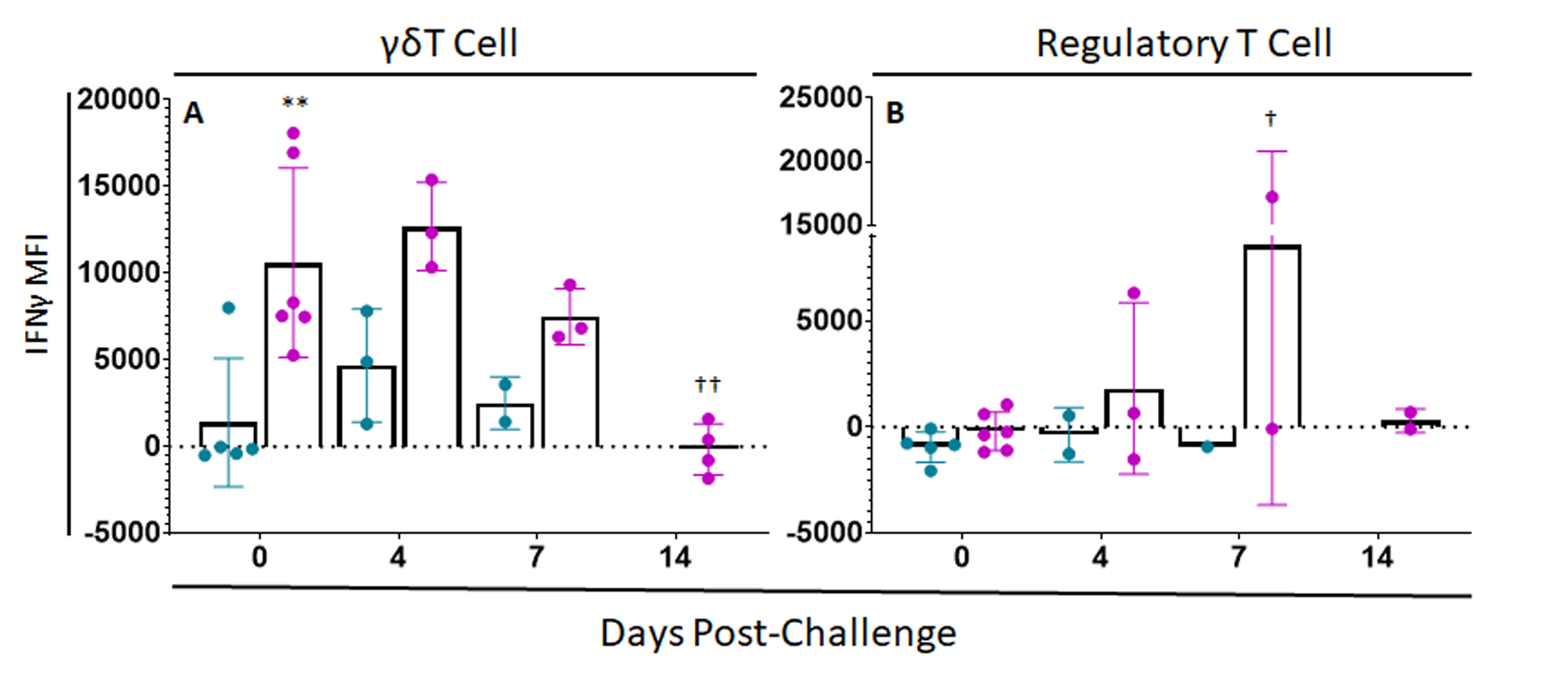

Supplement: Supplementary file 1 [file pathogens-11-01438-s001.zip › pathogens-1830988-supplementary/Supplemental material/Supplemental Figure S4 11.28.22.tif]
